# Ovarian Reserve Markers to Identify Poor Responders in the Context of Poseidon Classification

**DOI:** 10.3389/fendo.2019.00281

**Published:** 2019-05-08

**Authors:** Valentina Grisendi, Elisa Mastellari, Antonio La Marca

**Affiliations:** Section of Obstetrics and Gynecology, Mother-Infant Department, University of Modena and Reggio Emilia, Modena, Italy

**Keywords:** IVF, poor response, ovarian reserve markers, AMH, AFC, Bologna criteria, live birth rate, POSEIDON classification

## Abstract

It is well-known that poor ovarian reserve is a cause of infertility, poor response to gonadotrophin stimulation and poor success rate after *in vitro* fertilization (IVF) cycles. Some years ago a consensus was elaborated on precise criteria which can lead to a correct identification of poor responders (the Bologna criteria). More recently, the POSEIDON group has proposed a new stratified classification of patients with low prognosis, also with the aim of providing clinical indications for the management of these patients. A literature search was carried out for studies that investigated the ability of ovarian reserve markers, in particular AMH and AFC, to predict poor ovarian response in IVF cycles; secondly, studies regarding the Bologna criteria and their prognostic value were analyzed and available literature on POSEIDON classification was reported. The most recent markers of ovarian reserve (serum AMH and ultrasound AFC) have shown to provide a direct and accurate measurement of ovarian follicle pool. These markers have generally shown comparable predictive power for ovarian response and a number of retrieved oocytes in IVF cycles. “Abnormal ovarian reserve test” is a very important parameter both in the Bologna criteria and in the POSEIDON classification. Several studies have already been published about the reproductive outcome of patients defined as poor responders according to the ESHRE Bologna criteria: all of them agree on the poor IVF outcome and low pregnancy rate of these patients. Instead, being the POSEIDON classification of very recent publication, the efficacy of the POSEIDON approach in improving management and outcomes of POR patients has yet to be tested and validated with future prospective clinical trials. Prediction of poor response may help clinicians choose the stimulation protocol with the aim of gaining patient compliance and cost reduction, and many efforts have been made by researchers in this sense, including the formulation of the Bologna criteria and of the POSEIDON classification, in which the ovarian reserve markers (AMH and AFC) play a fundamental role.

## Introduction

In the last decades, a high number of studies has been carried out on the possibility of measuring ovarian reserve through ovarian reserve markers. In reproductive medicine this is a leading field of research, as ovarian reserve markers hold an important diagnostic and prognostic value. It is well-known that a low ovarian reserve may be an important cause of infertility. Moreover, knowing the ovarian reserve of a single woman allows clinicians to predict individual response to controlled ovarian stimulation (COS) in IVF cycles: if a patient has, for instance, a low ovarian reserve, she will probably achieve a poor ovarian response after COS. This condition is characterized by a low number of growing follicles and low serum estradiol levels after exogenous gonadotropin stimulation, resulting in a poor oocytes retrieval and, often, in a poor reproductive outcome (1–5). However, among poor responder patients the prognosis may be influenced by other parameters, such as patient's age and the outcome of previous IVF cycles.

For this reason, the European Society of Human Reproduction and Embryology (ESHRE) consensus has recently established that a response can be defined as poor (POR) when at least two of the following three criteria are present: (i) advanced female age (ii) a previous POR (iii) an abnormal ovarian reserve test (ORT) or in the absence of the above criteria two previous POR following maximal stimulation (6).

In literature, several studies have already been published on IVF outcome in poor responder patients defined according to the Bologna criteria. They all confirm a low live birth rate in these patients (7–9).

Nevertheless, since a certain heterogeneity concerning some biological and clinical features among patients included in the definition of POR still persists, a new classification stratified in subgroups based on these characteristics as well, that is the POSEIDON classification, was proposed in order to improve the performance of tailored therapies in the outcome of these patients and identify more homogeneous populations to be included in future clinical trials (10). This review aims at analyzing the role of ovarian reserve markers (AMH and antral follicle count) in predicting poor response after COS according to both classifications. Moreover, we will discuss the ability of the Bologna criteria to predict a poor reproductive prognosis in these patients as well as the innovations introduced by the POSEIDON classification.

## Definition of Ovarian Reserve Markers

Over the years, numerous ovarian reserve markers have been proposed. Serum FSH, measured in early follicular phase (day 3–5 of the menstrual cycle) together with estradiol, has been widely applied in reproductive medicine, but it is only an indirect marker of ovarian reserve and its blood concentrations rise only when ovarian reserve is severely compromised (11). Similarly, literature consistently reports only a moderate sensitivity and specificity of this marker in predicting ovarian response to ovarian stimulation. Various cut-off values ranging from 10 to 15 IU/L have been recommended for predicting poor response in IVF (12–15), but only few patients meet this high threshold, limiting the usefulness of the marker.

The most recent markers of ovarian reserve, i.e., serum AMH and ultrasound antral follicle count (AFC), have shown to provide a direct and accurate measurement of ovarian follicle pool. AMH is produced only by small antral follicles until 6–8 mm diameter and secreted in serum. AFC is performed by ultrasound and counts all identifiable antral follicles of 2–10 mm present in both ovaries (16–18). As the pool of small antral follicles measured when performing AFC is the same that secretes AMH and it is proportional to the overall number of primordial follicles in the ovaries, AFC and AMH are highly correlated and show similar values in reflecting oocyte quantity (19).

Comparing AMH with AFC, AMH has the advantage of a very little intra- and inter-cycle variability (20, 21). On top of the well-known age-related decline in AMH, significant fluctuations have been reported for a number of conditions and this has to be taken into account when interpreting values in clinical practice (20).

Fluctuations in the menstrual cycle have also been reported (22, 23), questioning about using AMH as a single reliable marker in the clinical situation. But these fluctuations appear to be random and minor, and limited to high AMH values, therefore not causing changes in the management of the single patient. This suggests that in clinical practice AMH can be measured independently of the cycle phase. The exact role of patients' characteristics like ethnicity or lifestyle, for example habits like smoking, on intra- and inter-individual variability of AMH needs to be further investigated. Moreover, problems of low comparability of measured values among laboratories related to the old manual essays seem nowadays to be solved by the new recent automated essays, which should guarantee repeatable and comparable dosages (24). The new automated essays measure lower AMH concentrations than the old manual essays (−16% with Access AMH and −20% with Elecsys AMH), but AMH levels are still strongly correlated to AFC, especially in patients with low ovarian reserve (24, 25).

On the other hand, AFC exhibits some degree of intra- and inter-cycle and inter-observer variability (21, 26) that must be taken into account when considering this marker for diagnostic purpose. In order to reduce such variability, recommendations on the methodology and on the equipment setting have been given (18). The recent introduction of three-dimensional (3D) automated follicular tracking should decrease the above mentioned variability (27, 28), but it needs advanced ultrasound equipment to date, which is not yet largely available.

Direct comparisons between AFC and serum AMH in IVF cycles have generally shown a similar capacity in predicting ovarian response and the number of retrieved oocytes. Having failed in showing an independent relationship between AFC and oocyte yield, a few studies are in favor of AMH as the strongest predictor of ovarian response, while other studies have proven AFC to have a stronger predictive value (29–35). Anyway, these markers globally predict ovarian response in IVF better than all other known markers (17, 36–39)

When introducing ovarian reserve markers in clinical practice with the objective of predicting ovarian response, it is fundamental to establish acceptable cut-off levels for the markers themselves. AMH and AFC cut-off values reported in literature are very variable (4). Such variability could be explained by factors such as the low number of subjects included in some of these studies, the variability in the measuring methods used for the two markers and the different definitions of poor ovarian response adopted by the various authors, consequently resulting in variations in the diagnostic performance of markers of ovarian reserve. Clinicians should therefore adopt cut-off values from the published study that may better reflect their clinical setting.

According to published studies, having good sensitivity and specificity, a cut-off value of AMH ranging between 0.7 and 1.3 ng/ml may be considered acceptable for the prediction of poor response in IVF (4, 37, 38). Using the most recent AMH automated essay (Access AMH), the cut-off point at 90% specificity and 74.1% sensitivity for poor response prediction was defined 0.93 ng/ml with ROC analysis (25).

AFC can be used to reliably predict ovarian response in IVF too, but literature shows a considerable variability in agreed AFC cut-off levels (17, 40). Inevitably, AFC thresholds for clinical practice depend on available ultrasound technology and resolution, and therefore need updating over time. Focusing on the most recent papers, generally based on modern technologies, the most frequently reported cut-off values for prediction of poor response range between AFC <5 and <7 (17). AFC thresholds calculated through modern 3D ultrasound technology haven't been published yet.

Thanks to their ability to predict ovarian response to stimulation, both markers are valid tools for the individualization of ovarian stimulation treatment and, in particular, for the choice of the starting dose of FSH (3, 4, 19, 32). A recent Cochrane review has confirmed that tailoring the FSH starting dose on ovarian reserve markers may reduce cases of OHSS, but it has not been able to demonstrate that it improves live birth rates compared to a policy of giving all women 150 IU (41). A recent multicenter prospective cohort study with two embedded RCTs, performed on 1,515 women randomized to an individualized or standard FSH dose (150 IU), reported the same conclusions: individualized dosing reduced the incidence of mild and moderate OHSS, but live births between the two groups were comparable (42).

Studies on the ability of AMH and AFC to predict oocytes quality and live births are controversial (32, 43–47). A recent study by La Marca et al. (48) reported a strong positive age-independent relationship between circulating AMH and the rate of euploidy in blastocysts after an IVF cycle (48). According to this observation, a large cohort study on 1230 IVF-ICSI cycles reported that AMH and age equivalently predict live birth (44). On the other hand, a large retrospective study performed on 69,336 fresh and 15,458 frozen embryo transfer cycles demonstrated that the areas under the curve (AUC) for AMH as predictor of live birth in fresh cycles and thawed cycles were, respectively, 0.631 and 0.540, suggesting that AMH alone is a weak, even if significant, age-independent predictor of live birth after ART (46).

## Ovarian Response Prediction and Management

The occurrence of poor ovarian response in IVF ranges from 10 to 20% and it increases with female age. Once a patient at risk of poor ovarian response is identified, she should be informed by the clinician about dramatic prospects. First of all, she will probably have a poor response to COS, which means a low number of oocytes retrieved following a standard IVF protocol. This results in a poor reproductive prognosis, as the chance of an adequate number of good embryos to transfer is reduced. Moreover, the clinician should inform the patient that the scientific community is, so far, not aware of any valid solution, that is of any therapeutic protocol that could modify or improve the patient's ovarian response and prognosis.

This information may be quite stressful for the patient, so it is necessary to accurately identify poor responder patients through standardized criteria. However, literature highlights that this is far from easy: in 2011 a systematic review (49) revealed that among 47 randomized trials on poor ovarian responders, 41 different definitions for women with poor ovarian response have been used, with differences both in the criteria considered for the definition (including age, ovarian reserve markers, outcomes of a previous IVF cycle like number of follicles on the last day of ovarian stimulation or number of oocytes retrieved, etc…) and in the threshold values used for each criteria.

The lack of uniformity in the definition of poor responder patients has convinced The European Society of Human Reproduction and Embryology (ESHRE) to elaborate a consensus where precise criteria lead to the identification of different groups (or “phenotypes”) of poor responders (6). In order to define a poor ovarian responder (POR), at least two of the following three features must be present: (i) Advanced maternal age (≥40 years) or any other risk factor for POR; (ii) A previous POR (≤3 oocytes with a conventional stimulation protocol);(iii) An abnormal ovarian reserve test (i.e., AFC <5–7 follicles or AMH <0.5 −1.1 ng/ml). In the absence of advanced maternal age or abnormal ORT, two episodes of POR after maximal stimulation are sufficient to define a patient as a poor responder. In particular, different categories of POR may be defined from the different combinations of the Bologna criteria as follows: (i) one previous poor response and age ≥40 years, (ii) one previous poor response and abnormal markers of ovarian reserve, (iii) age≥40 years and abnormal markers of ovarian reserve (the so-called expected poor response category), (iv) one previous poor response in a woman aged≥40 years and with abnormal markers of ovarian reserve, v) two previous POR cycles following maximal stimulation.

This classification recognizes that the reproductive prognosis and therefore the definition of POR certainly depends on the ovarian reserve, measured by AMH or AFC, but also on other anamnestic and clinical factors, such as age and outcome of a previous IVF cycle or previous ovarian surgery, which are integrated into the definition.

Following the consensus, several studies have been published about the reproductive outcome of patients defined as poor responders according to the ESHRE Bologna criteria. All of these studies agree on the poor IVF outcome and low pregnancy rate of these patients (7–9, 50). In a study by La Marca (7) a database containing IVF reports from 210 women defined as POR was analyzed. The study demonstrated that the five different groups of POR had similar IVF outcomes, with a live birth rate between 5.5 and 7.4%. It was therefore concluded that poor responders in the five subgroups identified by the Bologna criteria represent a homogenous population (7). The same results were reported in a retrospective study by Busnelli et al. (8), performed on 362 POR undergoing IVF: live birth rate was 6% (95% CI: 4–9%), not significantly different in the different subgroups of POR (8). In line with previous studies, in a large retrospective study by Bozdag et al. (9) performed on 821 patients fulfilling the Bologna criteria, the live birth rates per started IVF cycle ranged from 2.3 to 8.7%. In contrast with previous studies, the subgroup of POR presenting AFC <7 and a previous poor ovarian response, defined as “young proven” PORs subgroup, was found to be associated with the most favorable live birth and implantation rates, compared to other subgroups, characterized by patients' age >40 and a previous poor response and/or AFC < 7 (9).

By better identifying the perimeter of patients to be considered as poor responders, the Bologna Criteria certainly represent an important step in the definition of POR and for the prediction of an IVF cycle, thus allowing clinicians to provide patients with improved counseling.

A few authors (10, 51–53) have, however, focused on clinical and biological aspects that would deserve greater consideration in the classification of poor patients (including the age-related oocyte quality and the genetic profile that conditions the ovarian sensitivity to the stimulation with the gonadotropins). These authors have highlighted how, with regard to these aspects, there is a persistent heterogeneity among POR patients and have criticized the lack of indication of differentiated management strategies for the different subgroups of patients (Table 1). This means that the same type of treatment may not be optimal for all the patients defined as POR, even when having a similar prognosis. While this may have a logical basis, it is far from being proved on a solid scientific ground made of well-designed multicenter trials. Up to now, there is not sufficient evidence for clinicians to recommend a particular therapeutic strategy resulting in improved live birth rate for poor responder women.

**Table 1:** Summary of the existing literature on Poseidon classification.

**Author**	**Publication**	**Type of article**	**Main finding**
Poseidon Group (Patient-Oriented Strategies Encompassing IndividualizeD Oocyte Number), Alviggi et al. (10)	Fertil Steril. 2016; 105(6):1452–3	Commentary	Definition of Poseidon categories:
			Group 1: Patients < 35 years with sufficient prestimulation ovarian reserve parameters (AFC ≥5, AMH ≥1.2 ng/mL) and with an unexpected poor or suboptimal ovarian response.
			Group 2: Patients ≥ 35 years with sufficient prestimulation ovarian reserve parameters (AFC ≥5, AMH ≥1.2 ng/mL) and with an unexpected poor or suboptimal ovarian response.
			Group 3: Patients < 35 years with poor ovarian reserve prestimulation parameters (AFC < 5, AMH < 1.2 ng/mL).
			Group 4: Patients ≥ 35 years with poor ovarian reserve prestimulation parameters (AFC <5, AMH <1.2 ng/mL).
Humaidan et al. (54)	F1000Res. 2016; 5:2911	Commentary	Definition of Poseidon categories and discussion as to why the new concept has been proposed
Haahr et al. (52)	Reprod Biol Endocrinol. 2018; 16(1):20.	Review	Discussion on how the treatment of the expected poor ovarian response patient should be individualized in all steps of ART, including the choice of GnRH analog, the gonadotropin type and dose, ovulation trigger, and the possible use of adjuvant therapies.
Esteves et al. (53)	Front Endocrinol (Lausanne). 2018; 9:461.	Review	Critical appraisal of the existing criteria that standardize the definition of POR and explanation of reasons for the development of the POSEIDON criteria.

Treatment with a GnRH antagonist protocol instead of a GnRH agonist protocol was proposed for these patients as it avoids the very deep suppression of endogenous FSH and LH concentrations in the early follicular phase at the stage of follicular recruitment, thus giving hope for a better egg retrieval. Some trials and meta-analyses actually showed that the GnRH agonist long protocol and the GnRH antagonist regimen are comparable in their efficacy for the outcome of IVF in poor responders (55–57).

In this regard, we think that if the standard agonist long protocol offers no benefits in poor reponder patients in terms of chance of pregnancy when compared to an antagonist protocol, the choice of the protocol should aim to improve patient compliance (58) in addition to cost reduction (59). This is possible with an antagonist protocol as it allows shorter duration of stimulation and reduced gonadotrophin consumption (4, 60).

In this context, a recent large randomized trial demonstrated the non-inferiority of a mild ovarian stimulation protocol with GnRH antagonist compared to a standard approach with the long GnRH agonist protocol. Ongoing pregnancy rate was 12.8% (25/195) for mild ovarian stimulation vs. 13.6% (27/199) for conventional ovarian stimulation (95% CI: 0.57–1.57), while the duration of ovarian stimulation and the amount of gonadotrophins used were significantly lower in the mild stimulation strategy (61).

Different studies performed on women predicted to be poor responders showed that increasing the FSH dose doesn't impact on the number of retrieved oocytes (62, 63). The maximum number of oocytes that can be retrieved in women is determined by the number of recruitable antral follicles in the ovaries and it is obvious that a dose of FSH higher than the maximal one will never compensate for the lack of substrate.

Recently, new ovarian stimulation protocols are under study for their application on poor responders. Among these, the double ovarian stimulation, that is the ovarian stimulation in follicular and luteal phases within a single ovarian cycle, seems capable of increasing the number of retrieved oocytes and available embryos for the single patient (64). The first data have been published on possible advantages of a GH co-treatment with the mild stimulation protocol or the GnRH antagonist protocol in poor responders (65–67). Some RCTs and meta-analysis have also been published regarding LH supplementation within the rFSH stimulation in IVF cycles: there is no agreement on the benefit of this therapy in the general population, while particularly in POR patients it showed an improvement of the clinical pregnancy rate and live birth rate (51, 52, 68, 69). Supplementation with androgens also seems to give some positive results in these patients, although the available trials are still too low in number to make recommending such a therapy possible (52). Finally, a few studies report that a dual trigger ovulation regimen with GnRH agonist plus hCG could significantly improve number and maturity of retrieved oocytes in poor responders, but more studies are needed to evaluate a possible positive effect of the dual trigger on the clinical pregnancy rate (70, 71).

In this context, in order to differentiate more homogeneous subgroups of patients that could benefit from a specific management, in 2016 Alviggi and the POSEIDON group attempted to develop a new classification in which patients defined as “low prognosis” were divided in four groups according to age, ovarian reserve and ovarian response (Table 2). In the POSEIDON classification a poor ovarian reserve pre-stimulation is defined on the basis of ovarian reserve markers, precisely AFC <5 or AMH <1.2 ng/ml. This identifies the so-called “expected poor ovarian responders”: these patients belong to GROUP 3 if they are aged < 35 years or GROUP 4 if they are > 35 years old; the 2 groups with the same low ovarian reserve are thus differentiated for the oocyte quality and therefore for the expected aneuploidy rate of the oocytes taken. As a matter of fact, there is in literature a quite broad agreement that 35 years of age represent the beginning of age-related changes not only in oocytes quantity, but also in their quality (with an embryo euploidy rate that decreases by 2.4 percentage points for every year increase in female age and a blastocyst euploidy rate that drops from 60% before 35 years to 30% after 40 years, and a subsequent decline in implantation potential) (54, 72). In a subsequent review (52) the suggested recommendations for these categories of patients have been better explained: the number of oocytes necessary to obtain at least one euploid embryo for transfer in each patient is estimated between 4 and 7 for group 3 and 12 oocytes for group 4; suggested treatments in both groups include both the long GnRh agonist protocol and the GnRh antagonist protocol, ovarian stimulation with 300 IU/day of rFSH, with rLH adjuvant therapy for group 4, the possibility of performing a double stimulation with an oocyte/embryo accumulation and frozen embryo transfer. The use of an androgen adjuvant therapy (DHEA, testosterone) needs further investigation before it can be recommended whereas the possibility of oocyte donation is only considered in group 4.

**Table 2:** Comparison between Bologna criteria and Poseidon's stratification.

**Bologna criteria**	**Poseidon's stratification**
1) Maternal age ≥40 years + A previous POR	GROUP 1
	Age <35 years +
	adequate ORT +
	An unexpected POR (<9 oocytes retrieved)
2) An abnormal ORT (AFC <5–7 follicles or AMH < 0.5 −1.1 ng/ml) + A previous POR	GROUP 2
	Age ≥35 years +
	adequate ORT +
	An unexpected POR (<9 oocytes retrieved)
3) Maternal age ≥40 years + An abnormal ORT (AFC <5–7 follicles or AMH <0.5 −1.1 ng/ml)	GROUP 3
	Age <35 years +
	An abnormal ORT (AFC <5; AMH <1.2 ng/ml)
4) Maternal age ≥40 years + An abnormal ORT (AFC <5–7 follicles or AMH < 0.5 −1.1 ng/ml) + A previous POR	GROUP 4
	Age ≥35 years +
	An abnormal ORT (AFC <5; AMH <1.2 ng/ml)
5) 2 previous POR	

GROUPS 1 and 2 of the POSEIDON classification include instead the “unexpected poor ovarian response,”i.e., those patients who, despite having a good ovarian reserve based on the values of the ovarian reserve markers (AFC> 5, AMH> 1.2 mg / ml), obtained a low oocyte number: between 4 and 9 oocytes retrieved (subgroups 1-a and 2-a) or even fewer than 4 oocytes retrieved (subgroups 1-b and 2-b). Once again, given the same ovarian response, the two groups are distinguished according to the age of the patients (group 1 <35 years, group 2> 35 years). A poor response in these patients may be due to iatrogenic hypostimulation or also to genetic polymorphism in FSH receptors (FSHR), LH receptor (LHR) or LH. It is actually reported in the literature that some polymorphisms in the alleles coding for these molecules (such as the FSHR Ser680 allele, or the LHβ variant) are associated with higher FSH consumption, thus characterizing patients requiring higher doses of rFSH during the controlled ovarian stimulation for ART (52, 73, 74). At the current state of knowledge, no hormonal or ultrasound marker exists that allows for the early recognition of these polymorphisms in the single patient. Furthermore, an adjuvant therapy with rLH supplementation is suggested in this subgroup to increase the implantation rate and in group 2 to improve the oocyte quality also (68). Finally, it seems that genetic polymorphisms in FSH receptor are not associated to significant variation in ovarian reserve markers (75, 76).

Obviously the efficacy of the POSEIDON approach in improving management and outcomes of POR patients will have to be tested and validated with future prospective clinical trials. Moreover, AMH and AFC cut-off values for poor and normal response prediction will probably be updated in the next years, together with the spread of new AMH automated essays end modern ultrasound technology. As a consequence, POSEIDON categories might undergo a revision in the inclusion criteria on the ground of ovarian reserve markers.

## Conclusions

Serum AMH and ultrasound AFC have shown to provide a direct and accurate measurement of ovarian follicle pool. These markers have generally shown a similar capacity to predict ovarian response and number of retrieved oocytes in IVF cycles.

In spite of this, the definition of a patient as poor responder appears heterogeneous in literature, also for the fact that not only the ovarian reserve, but also other clinical-anamnestic factors are important in determining a poor response to COS. This is not irrelevant if we consider the clinical and psychological implications of a POR diagnosis. The development of the Bologna Criteria and of the POSEIDON classification, combining the ovarian reserve markers with age, with the previous response to COS and with other risk factors for POR, aims at providing a significant help in the standardization of the criteria used for this diagnosis and in the improvement of these patients' clinical management. While waiting for studies conducted on the new criteria, we can say that, according to the data currently available in literature, the prediction of poor response and the use of consequently tailored treatment can have positive results in terms of patient compliance and cost reduction, but it does not seem to involve a relevant improvement in IVF outcome (60, 77).

## Author Contributions

AL, VG, and EM contributed in literature search and in writing the review.

### Conflict of Interest Statement

The authors declare that the research was conducted in the absence of any commercial or financial relationships that could be construed as a potential conflict of interest. The handling Editor and reviewer CA declared their involvement as co-editors in the Research Topic, and confirm the absence of any other collaboration.
